# The herpesvirus accessory protein γ_1_34.5 facilitates viral replication by disabling mitochondrial translocation of RIG-I

**DOI:** 10.1371/journal.ppat.1009446

**Published:** 2021-03-26

**Authors:** Xing Liu, Yijie Ma, Kathleen Voss, Michiel van Gent, Ying Kai Chan, Michaela U. Gack, Michael Gale, Bin He

**Affiliations:** 1 Department of Microbiology and Immunology University of Illinois College of Medicine, Chicago, Illinois, United States of America; 2 Center for Innate Immunity and Immune Disease, Department Immunology, University of Washington, Seattle, Washington, United States of America; 3 Florida Research and Innovation Center, Cleveland Clinic, Port Saint Lucie, Florida, United States of America; 4 Department of Microbiology, The University of Chicago, Illinois, United States of America; 5 Department of Genetics, Harvard Medical School, Boston, Massachusetts, United States of America; 6 Wyss Institute for Biologically Inspired Engineering, Harvard University, Boston, Massachusetts, United States of America; Wayne State University, UNITED STATES

## Abstract

RIG-I and MDA5 are cytoplasmic RNA sensors that mediate cell intrinsic immunity against viral pathogens. While it has been well-established that RIG-I and MDA5 recognize RNA viruses, their interactive network with DNA viruses, including herpes simplex virus 1 (HSV-1), remains less clear. Using a combination of RNA-deep sequencing and genetic studies, we show that the γ_1_34.5 gene product, a virus-encoded virulence factor, enables HSV growth by neutralization of RIG-I dependent restriction. When expressed in mammalian cells, HSV-1 γ_1_34.5 targets RIG-I, which cripples cytosolic RNA sensing and subsequently suppresses antiviral gene expression. Rather than inhibition of RIG-I K63-linked ubiquitination, the γ_1_34.5 protein precludes the assembly of RIG-I and cellular chaperone 14-3-3ε into an active complex for mitochondrial translocation. The γ_1_34.5-mediated inhibition of RIG-I-14-3-3ε binding abrogates the access of RIG-I to mitochondrial antiviral-signaling protein (MAVS) and activation of interferon regulatory factor 3. As such, unlike wild type virus HSV-1, a recombinant HSV-1 in which γ_1_34.5 is deleted elicits efficient cytokine induction and replicates poorly, while genetic ablation of RIG-I expression, but not of MDA5 expression, rescues viral growth. Collectively, these findings suggest that viral suppression of cytosolic RNA sensing is a key determinant in the evolutionary arms race of a large DNA virus and its host.

## Author summary

Host cytosolic RNA sensing has been implicated in the recognition of herpesvirus infection. As such, herpesviruses likely have evolved strategies to escape this host surveillance mechanism; however, molecular insight into antagonism of RNA sensors by herpesviruses remains largely elusive. We show that the γ_1_34.5 protein encoded by herpes simplex virus 1 inactivates the helicase RIG-I that serves as an RNA receptor. Viral γ_1_34.5 hijacks RIG-I and selectively inhibits its engagement with the chaperone protein 14-3-3ε in the cytoplasm of infected cells. Targeting of RIG-I by γ_1_34.5 blocks the cytosol-to-mitochondrial translocation of RIG-I, which ultimately dampens antiviral innate immunity. Finally, depletion of RIG-I enhances the growth of a recombinant HSV-1 in which γ_1_34.5 was deleted. Our work provides insights into viral modulation of intracellular RNA recognition in herpesvirus infection, which in turn may guide the rational development of therapeutic agents.

## Introduction

The RIG-I-like receptors (RLR), which include RIG-I (retinoic acid-inducible gene-I), MDA5 (melanoma differentiation-associated gene 5), and LGP2 (laboratory of genetics and physiology 2), are best known to recognize RNA viruses [1,2]. Once bound to double-stranded RNA ligands, RLRs recruit mitochondrial antiviral-signaling protein (MAVS) to activate nuclear factor **κ**-B (NF-**κ**B) and interferon (IFN)-regulatory factor 3 (IRF3). Whereas LGP2 regulates innate immune signaling, RIG-I and MDA5 mediate the production of IFN-**α**/**β**, other cytokines and IFN-stimulated genes (ISGs).

The RIG-I protein consists of two caspase activation and recruitment domains at its N terminus, a helicase domain and a C-terminal domain [3]. Whereas RIG-I is in a closed, inactive conformation in uninfected cells, it adopts conformational changes upon activation by RNA viruses; this, then triggers RIG-I dephosphorylation, its K63-linked ubiquitination and mitochondrial translocation [2,4]. In this process, protein phosphatase 1, the ubiquitin ligases TRIM25 and Riplet, and the chaperone protein 14-3-3ε cooperatively allow RIG-I to active downstream targets, resulting in an antiviral state [5–8]. Emerging evidence also pinpoints to a role of RIG-I in the detection of DNA viruses [2,9]. RIG-I recognizes multiple RNAs of Kaposi’s sarcoma-associated herpesvirus (KSHV) and host-derived 5’-ppp-vtRNAs, which triggers IFN production and limits KSHV reactivation from latency [10–12]. In latently infected B cells, RIG-I binds to small noncoding RNA encoded by Epstein-Barr virus (EBV) to drive IL-10 production [13,14]. Remarkably, herpes simplex virus 1 (HSV-1) triggers RIG-I activation via RNA polymerase III that generates 5’-ppp RNA species, including host 5S ribosomal pseudogene transcripts due to virus-mediated depletion of specific RNA-binding proteins [15,16].

HSV-1 is an alphaherpesvirus associated with recurrent orofacial infection and remains the most common cause of viral encephalitis [17]. Upon infection, over 90 viral proteins are synthesized sequentially to facilitate viral persistence. Among them is the accessory protein **γ**_1_34.5 that is conserved between HSV-1 and HSV-2 [18–20]. The **γ**_1_34.5 protein critically mediates viral replication and penetration of the nervous system [18–20]. Accordingly, deletion of the **γ**_1_34.5 gene renders the virus avirulent, which is recently exploited for oncolytic HSV immunotherapy in humans [21–23]. How HSV-host interactions influence oncolytic HSV immunotherapy is largely unknown [24].

It has long been known that HSV-1 prevents translation arrest by double-stranded RNA-dependent protein kinase (PKR) through **γ**_1_34.5 [19,25,26]. Paradoxically, PKR inhibition *per se* does not restore viral virulence [27,28]. Previous work suggested that **γ**_1_34.5 is involved in HSV glycoprotein processing as well as nuclear egress [29–31]. Although the **γ**_1_34.5 protein suppresses autophagy [32], it appears dispensable for virus replication in non-neuronal cells where autophagy plays an important role in virus restriction [33]. HSV-1 **γ**_1_34.5 also inactivates the stimulator of interferon gene (STING), which only partially rescues viral growth [34]. To unravel key mechanisms of viral replication, we performed global gene expression and genetic analyses. We show that HSV-1 subverts RIG-I-mediated cytosolic RNA sensing via the **γ**_1_34.5 protein. Mechanistically, **γ**_1_34.5 targets RIG-I, which precludes the assembly of RIG-I and the chaperone 14-3-3**ε** into a translocon complex necessary for RIG-I translocation from the cytosol to mitochondria. As such, HSV-1 **γ**_1_34.5 inactivates RIG-I, which promotes effective viral replication.

## Results

### HSV-1 regulates host genes linked to cytosolic RNA recognition

**Fig 1:**
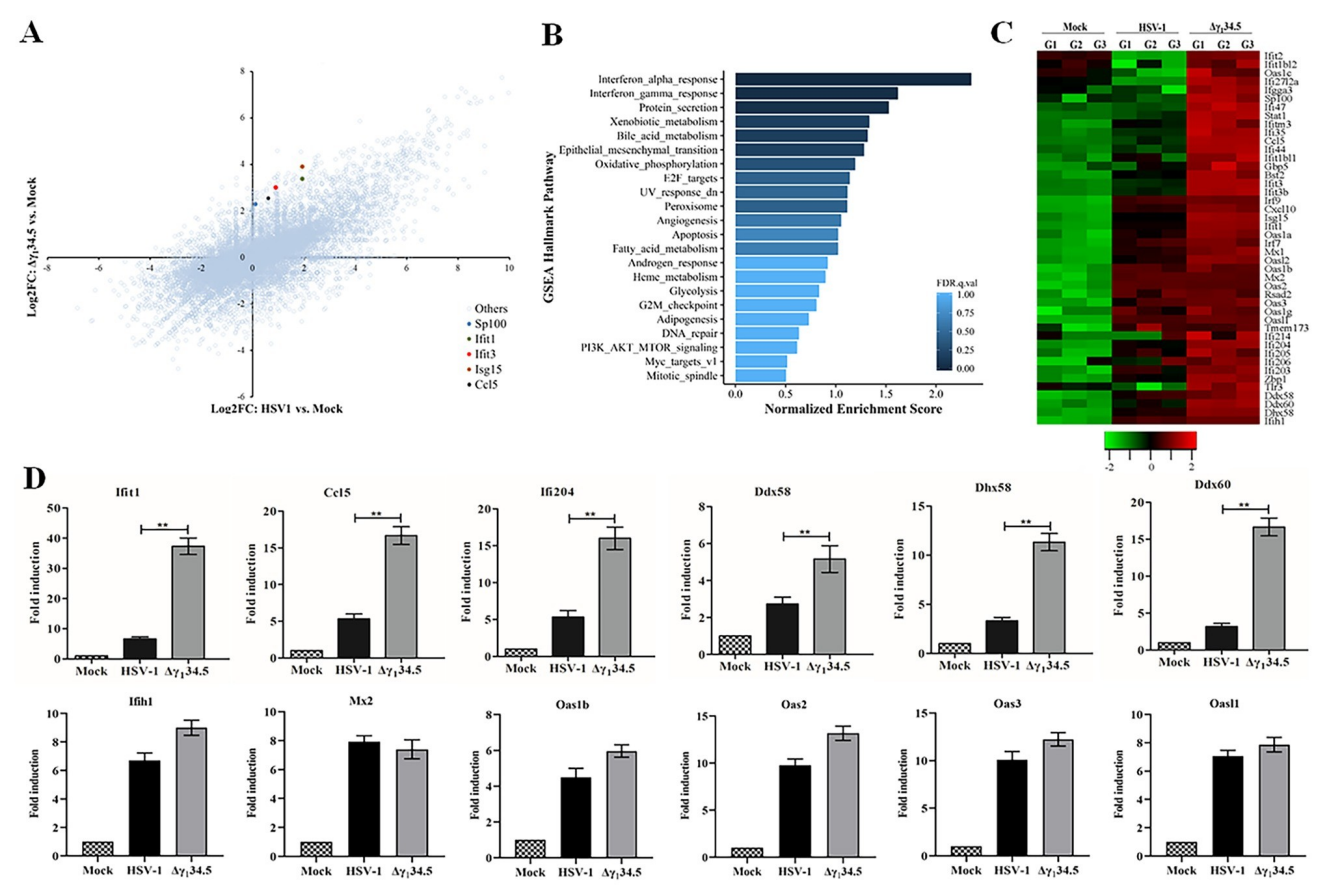
HSV-1 γ_1_34.5 downregulates immune genes linked to RNA sensing. (A) Effects of γ_1_34.5 on global host gene expression. MEF cells were mock infected, infected with wild type HSV-1 or Δγ_1_34.5 (5 pfu/cell). At 8 h postinfection, RNAs were extracted for RNA deep sequencing. Data from triplicate samples, processed as described in the Materials and Methods, are presented as Scatterplot. X axis denotes log2 fold change in HSV-1 to mock ratios. Y axis indicates log2 fold change in Δγ_1_34.5 to mock ratios. Dots are single genes. A few representative genes upregulated more by Δγ_1_34.5 than by wild type HSV-1 are highlighted. (B) GSEA hallmark analysis on genome wide gene expression. Pathways enriched in gene sets upregulated by Δγ_1_34.5 relative to wild type HSV-1 are ranked based on the normalized enrichment score (NES). False discovery rate (FDR) q value < 0.25 is defined as significantly enriched. Nominal p values are also indicated for top ranked pathways. (C) Heatmap visualization of RNA transcripts linked to the IFN response. The map shows 46 genes including IFN-stimulated genes, intracellular DNA sensors and RNA sensors. G1, G2 and G3 are distinct experimental replicates. The data represents the Log2FC (Fold Changes). (D) Effect of γ_1_34.5 on antiviral gene expression. MEF cells infected as in (A) were subjected to quantitative PCR analysis to test the expression of Ifit1, Ccl5, Ifi204, Ddx58, Dhx58, Ddx60, and Ifih1, Mx2, Oas1b, Oas2, Oas3, and Oasl1. The results were expressed as fold activation relative to 18S ribosomal RNA, with standard deviations among triplicate samples. The data were statistically analyzed by one-way ANOVA (**, *P* < 0.01).

To gain insight into HSV replication, we examined global gene expression in response to virus infection by RNA deep sequencing. Among diverse host genes, we observed a range of differentially up or downregulated ones by wild type HSV-1 and the γ_1_34.5 null mutant (Fig 1A). Several innate immune factors were also upregulated. Built on this initial assessment, we sought to define the functional pathways upregulated by the γ_1_34.5 null mutant relative to wild type virus. Gene Set Enrichment Analysis (GSEA) identified distinct pathways enriched (FDR q value < 0.25), which included the IFN-α/γ response, protein secretion, xenobiotic metabolism, bile acid metabolism, and epithelial mesenchymal transition (Fig 1B) [35].

Given the normalized enhancement scores closely coupled with the IFN pathways (Figs 1B and S1), we surveyed innate immune genes. This revealed unique patterns of RNA transcripts that were differentially accumulated (Fig 1C). We found that both viruses upregulated many IFN-related genes relative to the mock control, which reflects the cellular antiviral response. A more detailed comparative analysis of the genes upregulated by wild type vs mutant HSV-1 revealed several distinct features. The γ_1_34.5 null mutant, compared to wild type virus, highly stimulated a spectrum of IFN effector molecules, including Ifit1 (Isg56), Isg15, Sp100, Gbp5, Oasl2, Mx1, and Ifgga3. Moreover, As compared to the wild type virus, the γ_1_34.5 null mutant strongly increased transcript abundance of several DNA sensors (e.g. Ifi203, Ifi204, and Ifi205) that mediate antiviral gene induction in response to DNA ligands [36–38]. This data is in line with the fact that HSV-1 inactivates STING, a critical adaptor downstream of many DNA sensors, via the γ_1_34.5 gene product [34]. Importantly, the γ_1_34.5 null mutant exhibited propensity to induce more transcripts of Ddx60, Ddx58 (RIG-I), Dhx58 (LGP2), Ifih1 (MDA5) and Zbp1, which are prominent components of cytosolic RNA sensing pathways that regulate cytokine induction and necroptosis [1,39]. We also noted that wild type virus and the γ_1_34.5 null mutant comparably induced a subset of IFN-stimulated genes such as Mx2, Oas3 and Oasl1. These heterogeneous responses are likely attributable to a complex regulation of individual IFN-stimulated genes. We validated the RNAseq results by performing qPCR analysis of representative genes, which confirmed increased antiviral gene expression in the absence of γ_1_34.5 (Fig 1D). These results raised the possibility that besides intracellular DNA recognition, γ_1_34.5 might modulate cytosolic RNA sensing.

### The RIG-I-γ_1_34.5 axis influences the innate immune response

**Fig 2:**
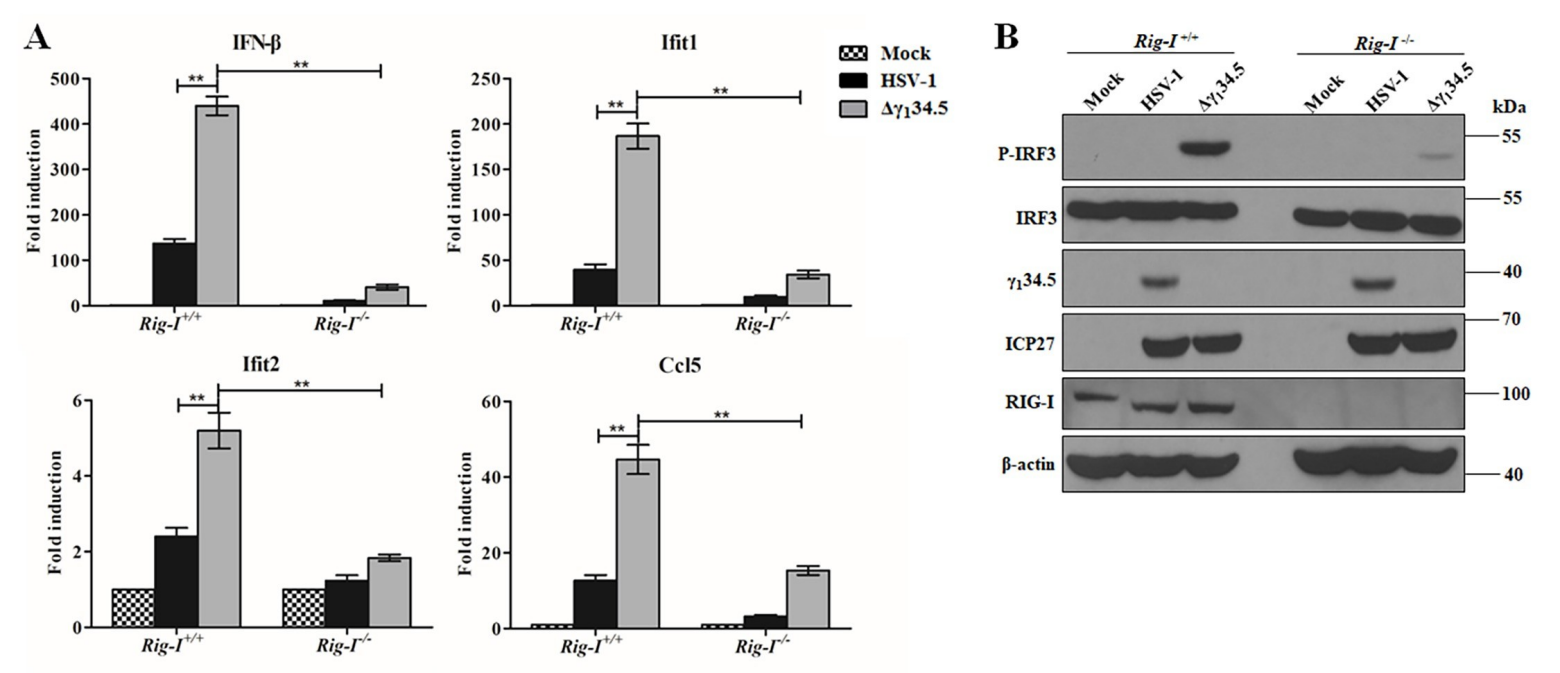
The γ_1_34.5 protein dampens antiviral responses mediated by RIG-I in MEF cells. (A) Effects of γ_1_34.5 on antiviral gene expression. *Rig-I*^*+/+*^ or *Rig-I*^*-/-*^ MEF cells were infected with wild type HSV-1 or Δγ_1_34.5 (5 pfu/cell). At 8 h after infection, RNA transcript levels of IFN-β, Ifit1, Ifit2, and Ccl5 were assessed by quantitative PCR analysis. The results were expressed as fold activation relative to 18S ribosomal RNA, with standard deviations among triplicate samples. The data were statistically analyzed by one-way ANOVA (**, *P* < 0.01). (B) Effects of γ_1_34.5 on IRF3 phosphorylation. *Rig-I*^*+/+*^ or *Rig-I*^*-/-*^ MEF cells were mock infected or infected with the indicated viruses (5 pfu/cell). At 8 h postinfection, cell lysates were processed for western blot analysis with antibodies against p-IRF3, IRF3, ICP27, γ_1_34.5, RIG-I, and β-actin. The experimental data are representative of results from three independent experiments.

**Fig 3:**
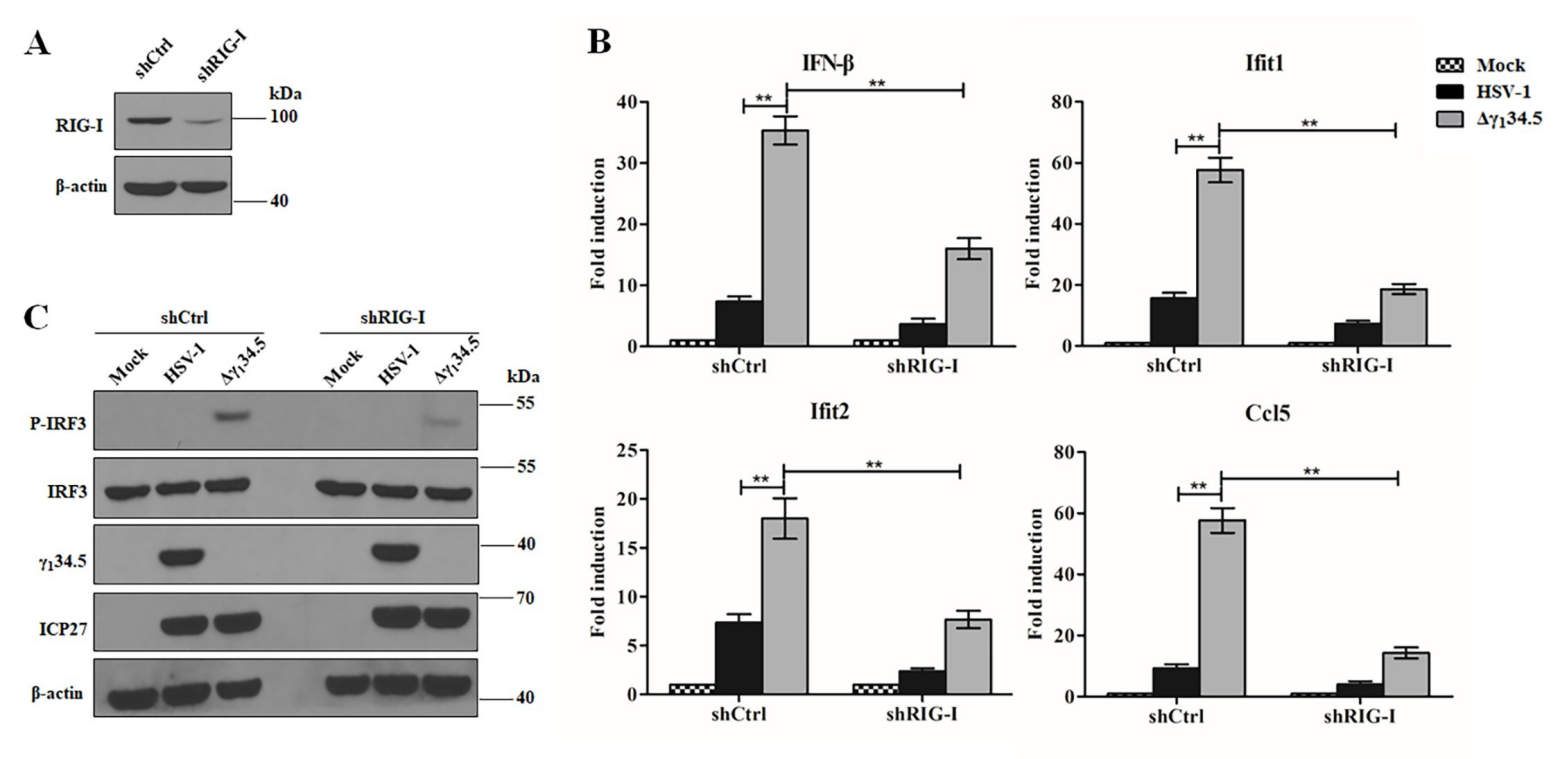
HSV-1 γ_1_34.5 reduces RIG-I dependent antiviral responses in human lung fibroblasts (HEL). (A) Validation of RIG-I knockdown in HEL cells. Cell lysates from HELs expressing control shRNA (shCtrl) or RIG-I target shRNA (shRIG-I) were subjected to western blot analysis with anti-RIG-I and β-actin antibodies. (B) Effects of γ_1_34.5 on antiviral gene expression in control or RIG-I knockdown HEL cells. Cells infected with wild type HSV-1 or Δγ_1_34.5 (5 pfu/cell) for 8 h were analyzed for transcript levels of IFN-β, Ifit1, Ifit2, and Ccl5 by quantitative PCR analysis. The data were statistically analyzed by one-way ANOVA (**, *P* < 0.01) with SD (n = 3). (C) Effects of γ_1_34.5 on IRF3 phosphorylation in shCtrl-transfected HEL or RIG-I knockdown HEL. Cells were infected as described in panel B and processed for Western blot analysis with antibodies against p-IRF3, IRF3, ICP27, γ_1_34.5 and β-actin. The experimental data are representative of results from three independent experiments.

Several lines of evidence show that HSV-1 triggers RIG-I to initiate antiviral signaling [2,9,15]. As wild type virus, unlike Δγ_1_34.5, favorably attenuated the IFN response, we reasoned that γ_1_34.5 might modulate innate immunity mediated by RIG-I. To probe this, we examined the induction of cytokines and ISGs by wild type HSV-1 and the γ_1_34.5 null virus in the presence or absence of RIG-I. In *Rig-I*^*+/+*^ mouse embryonic fibroblast (MEF) cells, wild type virus modestly induced the expression of IFN-β, Ifit1, Ifit2 (Isg54) and Ccl5 (RANTES) as measured by qPCR (Fig 2A). This was in stark contrast to the γ_1_34.5 null virus, which robustly induced the transcript expression of those genes, suggesting that the γ_1_34.5 protein acts to dampen RIG-I-dependent innate immune responses. Importantly, in infected *Rig-I*^*-/-*^ MEFs, viral induction of antiviral genes was greatly diminished, which attests a critical role of RIG-I in HSV-1 sensing as previously shown [15,16,40]. We further confirmed that a recombinant HSV, in which the γ_1_34.5 gene was restored, behaved like wild type virus, ruling out the possibility that the observed phenotypes were due to an irrelevant mutation(s) elsewhere in the virus genome (S2 Fig). To assess whether γ_1_34.5 functioned similarly in human cells, we determined cytokine expression in human lung fibroblasts infected with either wild type HSV-1 or the γ_1_34.5 null virus (Fig 3A and 3B). Albeit with a different magnitude, the γ_1_34.5 null virus readily induced expression of IFN-β, Ifit1, Ifit2 (Isg54) and Ccl5 (RANTES), relative to the wild type virus. RIG-I depletion by shRNA profoundly impaired such response to the HSV-1 variants. These results suggested that γ_1_34.5 dampens the antiviral response mediated by RIG-I.

To verify an effect of γ_1_34.5 on RIG-I signaling, we examined IRF3 phosphorylation, a hallmark of innate immune activation [1,41]. In *Rig-I*^*+/+*^ MEFs the γ_1_34.5 null virus, but not wild type virus, readily induced the phosphorylation of IRF3 (Fig 2B). Although viral infectivity, as measured by ICP27 expression, was comparable, phosphorylation of IRF3 occurred only with the virus devoid of γ_1_34.5 expression. The recombinant HSV-1 with repaired γ_1_34.5 inhibited IRF3 phosphorylation similarly to wild type HSV-1 (S2 Fig). In *Rig-I*^*-/-*^ MEFs virus-induced phosphorylation of IRF3 was abolished, further indicating a requirement of RIG-I in activating IRF3 during HSV-1 infection (Figs 2B and S2). Infection by the γ_1_34.5 null virus, but not wild type virus, also readily triggered IRF3 phosphorylation in human lung fibroblasts (Fig 3C). Taken together, these data show that the γ_1_34.5 gene product functions as a previously unrecognized herpesviral inhibitor of RIG-I-induced innate immune signaling.

### The γ_1_34.5 gene product interacts with and inhibits RIG-I

**Fig 4:**
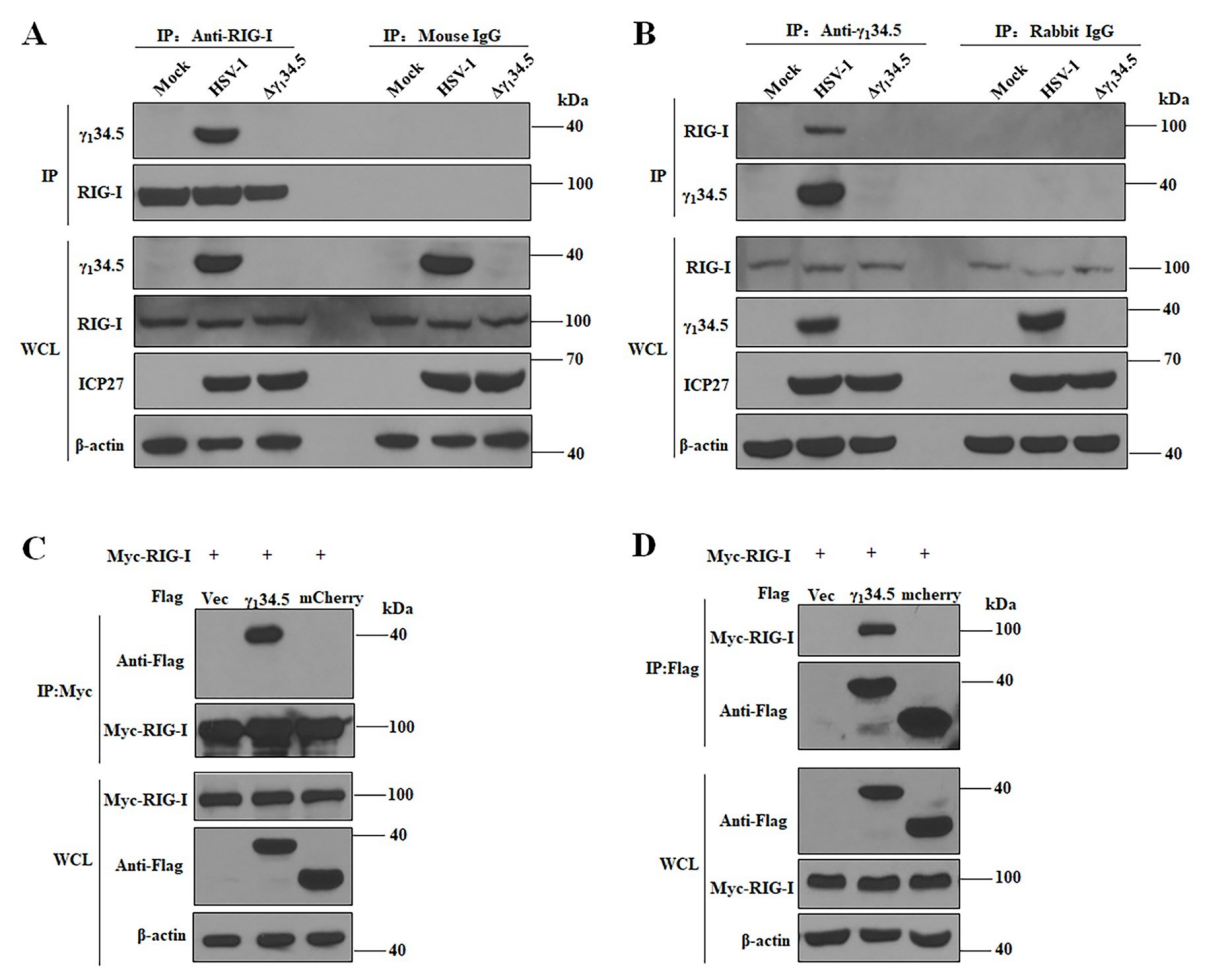
The γ_1_34.5 protein interacts with RIG-I. (A and B) The γ_1_34.5 protein interacts with endogenous RIG-I in infected cells. MEFs were infected with wild-type HSV-1 or Δγ_1_34.5 (10 pfu/cell). At 8 h postinfection, the cells were processed for immunoprecipitation (IP) with anti-RIG-I antibody or normal mouse IgG (A), or with anti-γ_1_34.5 antibody or normal rabbit IgG (B). Whole-cell lysates (WCL) and precipitated proteins were probed with antibodies against RIG-I, γ_1_34.5, ICP27 and β-actin. (C and D) HSV-1 γ_1_34.5 interacts with RIG-I in the absence of other viral proteins. HEK-293T cells were transfected with Myc-RIG-I together empty vector (Vec), Flag-γ_1_34.5 or Flag-mCherry for 36 h. Cell lysates were subjected to immunoprecipitation (IP) with anti-Myc antibody (C) or anti-Flag antibody (D). Precipitated proteins and WCLs were probed with antibodies against Flag, Myc, and β-actin. The data are representative of results from three independent experiments.

To determine whether γ_1_34.5 interacts with RIG-I, we performed immunoprecipitation using an anti-RIG-I antibody (Fig 4A). We found that γ_1_34.5 was precipitated with endogenous RIG-I in cells infected with wild type virus. Neither RIG-I nor γ_1_34.5 was precipitated by control IgG. The γ_1_34.5-RIG-I interaction was further verified in reciprocal immunoprecipitation with an anti-γ_1_34.5 antibody (Fig 4B). To determine whether γ_1_34.5 can bind RIG-I in the absence of other HSV proteins, we tested this interaction in 293T cells co-expressing Flag-γ_1_34.5 or Flag-mCherry (control) together with Myc-RIG-I. As shown in Fig 4C, HSV-1 γ_1_34.5, but not irrelevant mCherry, precipitated with Myc-RIG-I by IP with anti-Myc antibody. Conversely, RIG-I was specifically precipitated with γ_1_34.5 by IP with anti-Flag antibody (Fig 4D). Moreover, γ_1_34.5 interacted with the 2CARD domain of RIG-I and inhibited 2CARD-mediated IFN-β activation (S3 Fig). Deletion of the N- or C-terminal domain from γ_1_34.5 abrogated its ability to interact with RIG-I and inhibit RIG-I signaling (S4 Fig). These observations strongly suggest that the γ_1_34.5 protein inhibits cytosolic RNA sensing by targeting RIG-I in HSV-1 infection.

### The γ_1_34.5 protein blocks the mitochondrial translocation of RIG-I

**Fig 5:**
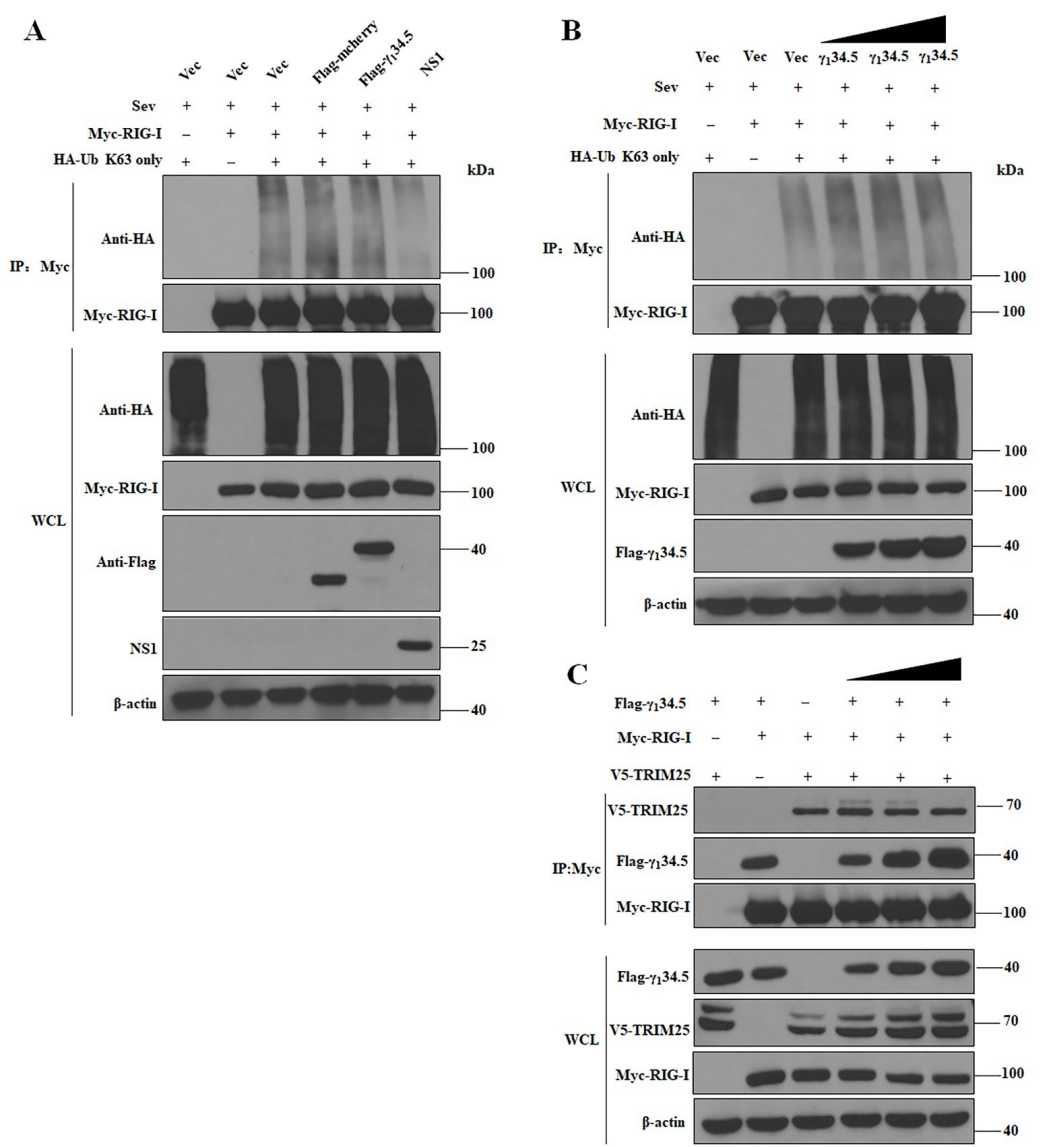
The γ_1_34.5 protein does not inhibit the K63-linked ubiquitination of RIG-I. (A) Effect of γ_1_34.5 on ubiquitination. HEK-293T cells were transfected with plasmids encoding Myc-RIG-I and HA-Ub (K63 only) together with empty vector (Vec), Flag-mCherry, Flag-γ_1_34.5 or pCAGGS-NS1 (positive control). At 24 h after transfection, the cells were treated with SeV (100 HA/ml) for additional 12 h and then harvested for ubiquitination analysis as described in the Materials and Methods. Whole-cell lysates (WCLs) were subjected to immunoprecipitation (IP) with anti-Myc antibody. Precipitated proteins and WCLs were probed with antibodies against HA, Flag, Myc, and β-actin. (B) Effect of different doses of γ_1_34.5 on RIG-I ubiquitination. HEK-293T cells were transfected with increasing amounts of Flag-γ_1_34.5 and assayed as described as in (A). (C) The γ_1_34.5 protein does not disrupt the interaction of RIG-I and TRIM25. HEK-293T cells were transfected with plasmids encoding Myc-RIG-I and V5-TRIM25 together with empty vector (Vec) or different doses of Flag-γ_1_34.5 for 36 h. WCLs were subjected to IP with anti-Myc antibody. Precipitated proteins and WCLs were probed with antibodies against Flag, Myc, V5, and β-actin. The experimental data are representative of results from three independent experiments.

**Fig 6:**
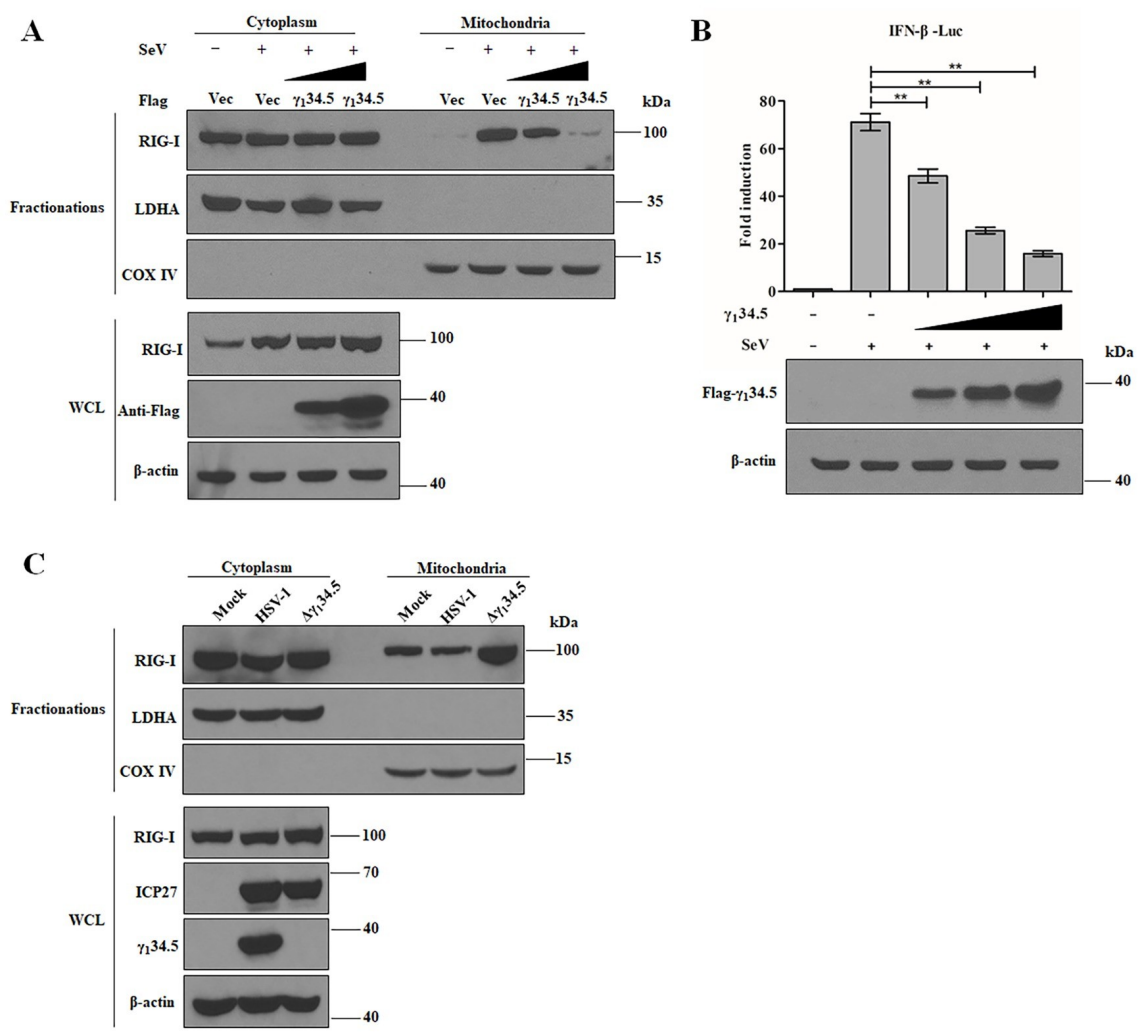
The γ_1_34.5 protein blocks RIG-I translocation to the mitochondria. (A) Ectopic expression of γ_1_34.5 inhibits RIG-I mitochondrial translocation in response to SeV infection. HEK-293T cells were transfected with empty vector (Vec) or increasing amounts of Flag-γ_1_34.5. At 24 h posttransfection, the cells were infected with SeV (100 HA/ml) for additional 24 h and harvested for cytoplasmic and mitochondrial fractionation. Samples were subjected to Western blot analysis with antibody against RIG-I, LDHA, COX IV and Flag. (B) The γ_1_34.5 protein inhibits IFN-β promoter activation by SeV. HEK-293T cells were co-transfected with pIFN-β-luc (50 ng) and pRL-TK (10 ng) along with either vector or Flag-γ_1_34.5 (range from 200 to 800 ng). At 24 h posttransfection, the cells were treated with SeV (100 HA/ml) for 24 h and harvested for luciferase assays. Results are expressed as means ± standard deviations (SD) (*n* = 3) and assessed by one-way ANOVA (**, *P* < 0.01). (C) Block of RIG-I mitochondrial translocation by HSV-1 requires γ_1_34.5. MEF cells were mock-infected or infected with wild type HSV-1 or Δγ_1_34.5 (10 pfu/cell). At 8 h post infection, whole lysates, cytoplasmic and mitochondrial fractions were collected. Samples were processed for Western blot analysis with antibody against RIG-I, LDHA, COX IV, γ_1_34.5 and β-actin. The experimental data are representative of results from three independent experiments.

To initiate antiviral immunity, RIG-I undergoes K63-linked ubiquitination by the E3 ligase tripartite motif-containing protein (TRIM25) and subsequently moves from the cytoplasm to mitochondria [1]. We noted that Sendai virus (SeV) effectively induced K63-linked ubiquitination of RIG-I ectopically expressed in 293T cells. Whereas influenza A virus NS1, a viral TRIM25 antagonist [42], potently diminished the K63-linked ubiquitination of RIG-I, HSV-1 γ_1_34.5 exhibited little inhibitory effect on RIG-I polyubiquitination (Fig 5A and 5B). Congruently, the γ_1_34.5 protein did not interrupt the interaction of TRIM25 and RIG-I (Fig 5C), suggesting a different mechanism is in operation. To test this, we examined the subcellular localization of RIG-I by fractionation analysis. In uninfected control 293T cells, RIG-I was seen primarily in the cytoplasm, as expected (Fig 6A). SeV infection markedly increased the abundance of RIG-I at the mitochondria; however, overexpression of γ_1_34.5 substantially reduced the abundance of RIG-I in the mitochondrial fraction, which correlated with reduced IFN-promoter activation (Fig 6B). These results demonstrate that the γ_1_34.5 protein interrupts the translocation of RIG-I from the cytoplasm to the mitochondria induced by RNA virus infection.

We next analyzed the cytosol-to-mitochondria re-localization of RIG-I in HSV-1-infected cells. RIG-I predominantly localized to the cytoplasmic fraction in mock-infected MEF cells (Fig 6C). Infection with the γ_1_34.5 null virus drastically increased the abundance of RIG-I in the mitochondrial fraction. However, this increase was not detectable in wild type HSV-1 infected cells. Taken in combination, these results show that the γ_1_34.5 protein prevents the mitochondrial translocation of RIG-I during HSV-1 infection.

### HSV-1 γ_1_34.5 prevents assembly of RIG-I and 14-3-3ε into a functional translocation complex

**Fig 7:**
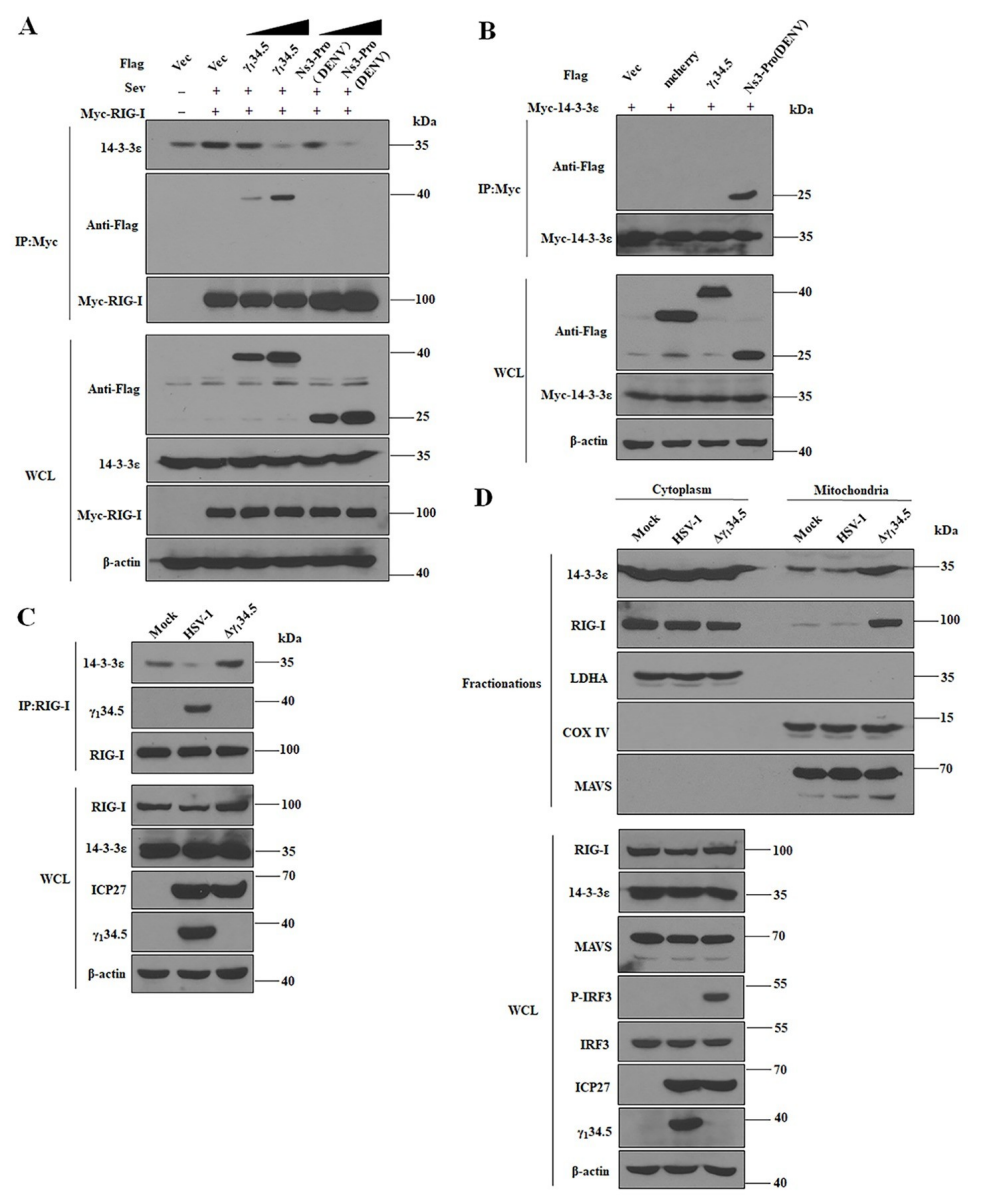
The γ_1_34.5 protein precludes formation of the RIG-I-14-3-3ε complex required for mitochondrial translocation and IRF3 activation. (A) The γ_1_34.5 protein prevents the interaction of RIG-I and 14-3-3ε. HEK-293T cells were transfected with Myc-RIG-I together with vector plasmid or Flag-γ_1_34.5 or lag-Ns3-pro (DENV). At 24 h posttransfection, cells were treated with SeV (100HA/ml) for 24 h. Whole-cell lysates (WCLs) were subjected to immunoprecipitation (IP) with anti-Myc antibody. Precipitated proteins and WCLs were probed with antibodies against Flag, Myc, 14-3-3ε and β-actin. (B) The γ_1_34.5 protein dose not interact with 14-3-3ε. HEK-293T cells were co-transfected with Myc-14-3-3ε along with vector plasmids, Flag-mCherry, Flag-γ_1_34.5 and Flag-Ns3-pro (DENV) for 36 h. Cells were then harvested and subjected to immunoprecipitation (IP) with anti-Myc antibody. Precipitated proteins and WCLs were probed with antibodies against Flag, Myc, and β-actin. (C) Inhibition of the RIG-I-14-3-3ε complex formation by HSV-1 requires γ_1_34.5. MEFs were infected with wild type HSV-1 or Δγ_1_34.5 (10 pfu/cell). At 8 h postinfection, cells were processed for immunoprecipitation (IP) with anti-RIG-I antibody. Whole-cell lysates and precipitated proteins were probed with antibodies against RIG-I, 14-3-3ε, γ_1_34.5, ICP27 and β-actin. (D) Block of RIG-I mitochondrial translocation by γ_1_34.5 inhibits IRF3 phosphorylation. MEFs were infected with wild type HSV-1 or Δγ_1_34.5 (10 pfu/cell). At 8h postinfection, cells were harvested for cytoplasmic and mitochondrial fractionation. Samples were processed for Western analysis with antibodies against 14-3-3ε, RIG-I, LDHA, COX IV MAVS, phosphorylated IRF3, IRF3, ICP27, γ_1_34.5 and β-actin. The data are representative of results from three independent experiments.

Cellular 14-3-3 proteins are essential components of cytosolic RNA sensing machineries [8,43]. In response to RNA virus infections, 14-3-3ε forms a complex with RIG-I to facilitate its mitochondrial translocation, activating downstream signaling. To further probe the mechanism of γ_1_34.5 action, we assessed the effect of γ_1_34.5 on RIG-I-14-3-3ε complex formation. Myc-RIG-I detectably bound to endogenous 14-3-3ε in mock-infected cells, and infection with SeV enhanced such interaction (Fig 7A). The weaker band in the sample with vector co-transfection likely reflected non-specific binding. However, the presence of γ_1_34.5 inhibited the binding of RIG-I to 14-3-3ε, as did dengue NS3, a viral antagonist of 14-3-3ε [44]. Interestingly, dengue NS3 bound to 14-3-3ε whereas γ_1_34.5 failed to do so (Fig 7B). We reasoned that the γ_1_34.5 protein may specifically target RIG-I, which prevents its assembly into the 14-3-3 translocation complex. To further test this, we analyzed 14-3-3ε-RIG-I binding in HSV-1-infected cells. As illustrated in Fig 7C, only a small amount of 14-3-3ε was precipitated with RIG-I in mock infected cells; however, infection with the γ_1_34.5 null virus increased the amount of 14-3-3ε that co-precipitated with RIG-I. Crucially, infection with wild type HSV-1 nearly eliminated the binding of 14-3-3ε to RIG-I, which coincided with γ_1_34.5 bound to RIG-I.

To determine the consequence of γ_1_34.5 expression on downstream signaling, we assessed IRF3 activation with respect to the distribution of 14-3-3ε and RIG-I (Fig 7D). We observed that in mock-infected cells, RIG-I and 14-3-3ε were predominantly localized to the cytoplasm. On the other hand, MAVS was seen at the mitochondria, as expected. Infection with the γ_1_34.5 null virus significantly increased the levels of 14-3-3ε and RIG-I at the mitochondria. However, this was not detectable with wild type virus, indicative of a block in mitochondrial translocation of RIG-I. Inhibition of RIG-I mitochondrial translocation also occurred upon ectopic expression of γ_1_34.5 (S5 Fig), indicating that γ_1_34.5 is directly responsible for this effect. Further analysis showed that unlike wild type HSV-1, the γ_1_34.5 null virus induced phosphorylation of IRF3 (Fig 7D). Thus, the γ_1_34.5 protein functionally disengaged RIG-I from 14-3-3ε to halt downstream signaling. This demonstrates that HSV-1 γ_1_34.5 specifically interrupts a key step of RIG-I activation.

### The RIG-I-γ_1_34.5 interaction affects viral growth

**Fig 8:**
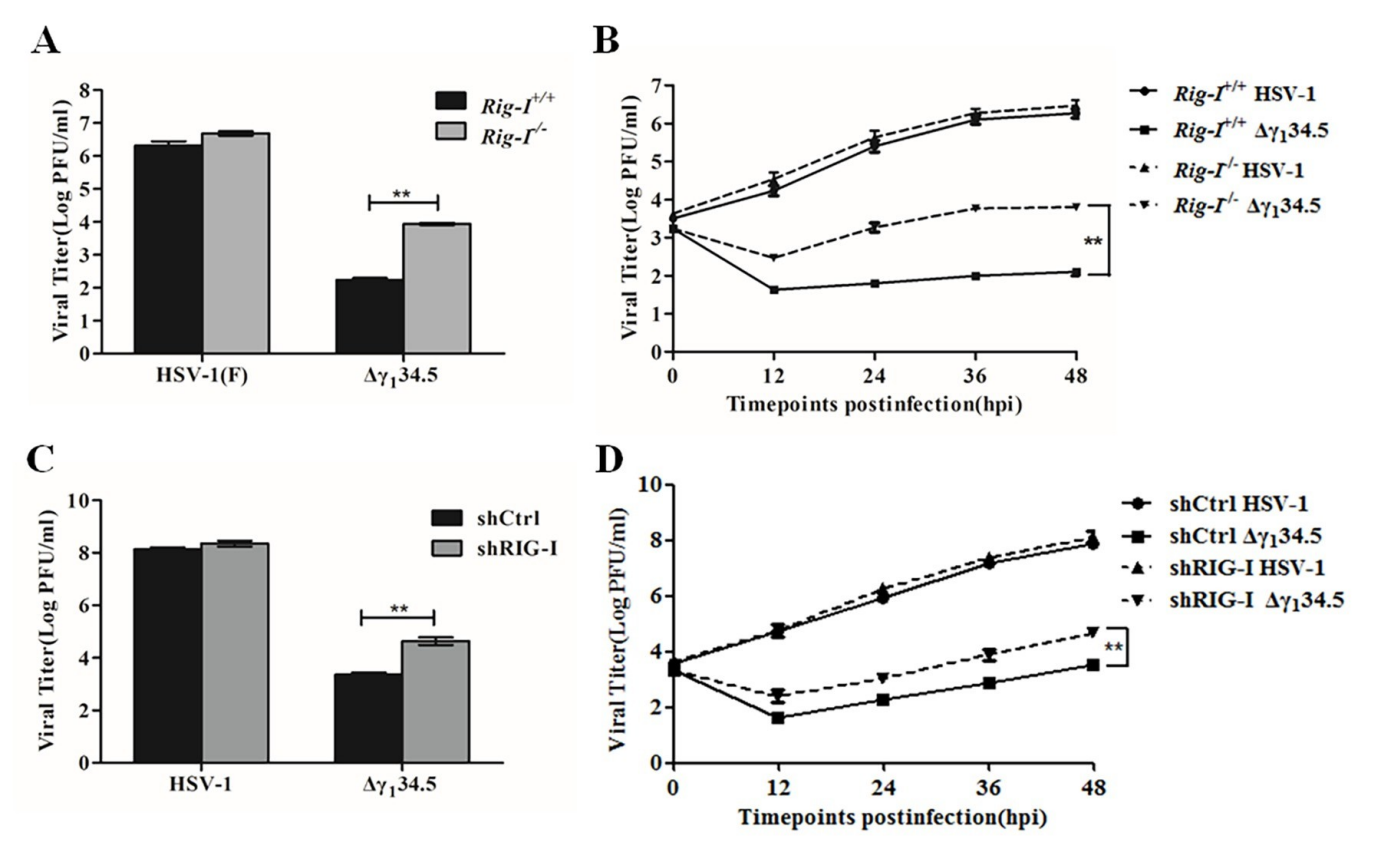
The γ_1_34.5-RIG-I interaction influences HSV-1 replication. (A) Viral replication in *Rig-I*^*+/+*^ or *Rig-I*^*-/-*^ MEFs. Cells were infected with wild-type HSV-1 or the γ_1_34.5 deletion virus (Δγ_1_34.5) at a MOI 0.01. At 48 h postinfection, virus yields were determined on Vero cells by plaque assay. (B) Kinetics of viral growth in *Rig-I*^*+/+*^ or *Rig-I*^*-/-*^ MEFs. Viral infection was performed as described for panel (A) and viral yields were measured at the indicated time points. (C) Viral replication in control and RIG-I knockdown human lung fibroblasts cells. shCtrl (control) or shRIG-I (RIG-I knockdown) HEL cells were infected with wild type HSV-1 or Δγ_1_34.5 (0.01 pfu/cell). At 48 h postinfection, virus yields were determined by plaque assay. (D) Kinetics of viral growth in control and RIG-I knockdown cells. Viral infection was performed as described in panel (C) and viral yields were measured at the indicated time points. The data are representative of results from three experiments with triplicate samples. Differences between the selected groups were statistically assessed by one-way ANOVA (A and C) or a two-tailed Student’s t test (B and D) (**, *P* < 0.01).

Lastly, we assessed the impact of the RIG-I-γ_1_34.5 interaction on viral growth. Fig 8A shows that wild type HSV-1 replicated robustly in both *Rig-I*^*+/+*^ and *Rig-I*^*-/-*^ MEF cells, with titers reaching 2 x 10^6^ and 4 x 10^6^ pfu/ml, respectively. In contrast, the γ_1_34.5 null virus replicated poorly in *Rig-I*^*+/+*^ MEF cells, with a titer of 2 x 10^2^ pfu/ml. The growth defect of the γ_1_34.5 null virus was dramatically restored in *Rig-I*^*-/-*^ MEF cells (1 x 10^4^ pfu/ml). We further examined the kinetics of viral growth (Fig 8B). In *Rig-I*^*+/+*^ MEF cells, wild type virus grew steadily as infection progressed, with a titer increasing to 1 x 10^6^ pfu/ml at 48 h post-infection. However, the γ_1_34.5 null mutant barely replicated in *Rig-I*^*+/+*^ cells throughout infection, with a titer of <1 x 10^2^ pfu/ml. In *Rig-I*^*-/-*^ cells, both viruses replicated more efficiently than each virus did in *Rig-I*^*+/+*^ cells, with a faster growth kinetics. Similarly, knockdown of RIG-I by shRNA enhanced HSV replication in human lung fibroblasts in the absence γ_1_34.5 (Fig 8C and 8D). In contrast, virus replication was comparable in the presence and absence of the related RNA sensor MDA5 (S6 Fig). Collectively, these results demonstrate that RIG-I functions to limit HSV-1 replication, where the γ_1_34.5 protein serves to overcome the RIG-I-mediated virus restriction.

## Discussion

Productive herpesvirus infection involves viral blockade of translation arrest by the dsRNA-dependent protein kinase, PKR [26,45–47]. Whether and how herpesviruses interact with the networks of cytosolic RNA sensors has been unresolved. Here we identified a previously unrecognized mechanism by which HSV-1 inhibits cytosolic RNA recognition by RIG-I. This activity is dependent on the virulence factor γ_1_34.5, which prevents assembly of RIG-I and 14-3-3ε into a ‘translocon’ complex, thereby impairing subsequent IRF3 phosphorylation and antiviral immunity. This work further underscores the importance of RIG-I in HSV-1 restriction [15,16,40].

As a large DNA virus, HSV-1 replication proceeds temporally, generating various virus- and host-derived stimulatory RNA species during the course of infection [15,17] that likely trigger several RNA sensing pathways simultaneously or sequentially. First, onset of HSV DNA replication is thought to activate PKR via dsRNA and shuts off protein synthesis [19]. Yet, viral γ_1_34.5, in cooperation with Us11, functionally inhibits PKR at discrete phases of HSV infection [48]. This involves dephosphorylation of the eukaryotic translation initiation factor eIF2 alpha by γ_1_34.5, which facilitates neuroinvasion *in vivo* [26,49]. Published work also indicates that herpesviruses instigate RIG-I through RNA polymerase III, a distinct pathway to initiate antiviral immunity [16,50]. HSV-1 infection causes translocation of host 5S ribosomal pseudogene transcripts (in particular RNA5SP141; also generated by RNA polymerase III) from the nucleus to the cytoplasm, and their subsequent unmasking by HSV-1-mediated depletion of RNA5SP141-binding proteins leads to activation of RIG-I [15]. RIG-I activation imposes another barrier to HSV-1 which would necessitate the immune-evasive actions of one or more viral genes to facilitate infection. As γ_1_34.5 is expressed early as well as late in infection [51–53], it may serve to control different RNA sensing machineries, which ensures the progression of HSV-1 replication.

We recently reported that the γ_1_34.5 protein interferes with the DNA sensing pathway through STING inactivation [34]. STING acts downstream of several DNA sensors, including cyclic GAMP synthase (cGAS), IFI16 and DDX41 that detect and limit HSV infection [36,54–56]. HSV γ_1_34.5 directly targets STING, and this interaction depends on the N-terminal domain of γ_1_34.5 [34]. This is different from its regulation of RIG-I, where full-length γ_1_34.5 is required for RIG-I antagonism as indicated by our data that showed that deletion of either the N-terminal or C-terminal domain of γ_1_34.5 abolished its activity against RIG-I. This suggests that the γ_1_34.5 protein functions to regulate two major signaling proteins in innate sensing (RIG-I and STING) using distinct interacting modes. In this context, it is notable that crosstalk between RIG-I and STING has been reported. For example, RIG-I activation by synthetic or viral agonists induces STING expression [57]. Conversely, STING deficiency leads to diminished IFN production in response to dsRNA or RNA virus infection [54,58]. Upregulated RIG-I can also participate in STING degradation [59]. Furthermore, temporally distinct roles have been reported for cGAS and RIG-I in the sensing of HSV-1 infection and subsequent cytokine induction [15]. Thus, a complex interplay between RIG-I and STING exists, and how γ_1_34.5 coordinately controls these two antiviral pathways throughout the HSV life cycle is an important question that awaits further investigation. Considering host selective pressures, our results suggest that the γ_1_34.5 gene of HSV-1 may have evolved to cope with RIG-I in addition to PKR and STING. This model may explain, at least in part, why the γ_1_34.5 protein functions as an HSV virulence factor *in vivo* [60], which warrants further investigation.

The mechanisms of RIG-I regulation are under intensive investigation [1,2]. Accumulating studies show that herpesviruses activate RIG-I via both viral and host-derived RNAs [10–12,15,16]. The mechanism by which HSV-1 γ_1_34.5 inactivates RIG-I has been unknown. We found that HSV-1 γ_1_34.5 displayed no inhibitory effect on the K63-linked ubiquitination of RIG-I mediated by TRIM25, an essential step in RIG-I activation [1,2]. Instead, γ_1_34.5 prevented the assembly of the RIG-I-14-3-3ε complex and its re-localization from the cytoplasm to the mitochondria. This illustrates a powerful mechanism by which HSV-1 avoids MAVS activation, IRF3 phosphorylation and subsequent cytokine expression. As intact γ_1_34.5 is required to interact with the CARDs of RIG-I, we infer that γ_1_34.5 may compete with 14-3-3ε for the binding site on RIG-I. Alternatively, HSV-1 γ_1_34.5 may alter the conformation of RIG-I that is required for access by 14-3-3ε, which then halts mitochondrial translocation of RIG-I. We propose that while RNA ligands induce conformational changes and posttranslational modifications of RIG-I, γ_1_34.5 serves to selectively disable the cytosol-to-mitochondria translocation of RIG-I, which ultimately inhibits immune activation.

Our work reveals that the γ_1_34.5-RIG-I interaction influences HSV-1 replication. In the presence of RIG-I, wild type virus replicated efficiently whereas deletion of the γ_1_34.5 gene crippled viral replication. This is linked to the ability of γ_1_34.5 to block the mitochondrial translocation of RIG-I, which phenotypically resembles the antagonistic activity by the NS3 proteins of dengue and Zika viruses [44,61]. Unlike the flavivirus NS3 proteins, HSV-1 γ_1_34.5 uniquely targets RIG-I instead of 14-3-3. Many other RNA viruses perturb the RIG-I signaling pathway [62]. For example, influenza A virus NS1 inhibits the K63-linked ubiquitination of RIG-I mediated by TRIM25 [42]. The NS3/4A protease of hepatitis C virus cleaves MAVS [63,64] and also abolishes RIG-I ubiquitination by Riplet [65], while the 3C proteins from certain picornaviruses cleave RIG-I [66]. Other studies suggest that the BPLF1 protein of Epstein-Barr virus (EBV) inhibits the K63-linked ubiquitination of RIG-I through recruitment of 14-3-3 to sequester and inactivate TRIM25, whereas ORF64 of KSHV directly cleaves K63-polyubiquitin chains of RIG-I [67]. HSV-1 UL37 deaminates and inhibits RIG-I sensing [68,69]. On the other hand, Us11 of HSV-1 impairs RIG-I via PACT inactivation [70,71]. Additional modulation of RIG-I by γ_1_34.5 indicates a complex regulatory circuit that might be relevant to temporal replication of herpesviruses. Strikingly, genetic ablation of RIG-I, but not MDA5, markedly reversed the HSV-1 growth defect in the absence of γ_1_34.5. Such specificity may reflect the requirement of a separate HSV function for restricting MDA5, or it may imply that MDA5 is not required for the restriction of HSV-1 replication. Further work is required to clarify this issue.

It is noteworthy that inborn errors in type I IFN-mediated immunity contribute to HSV encephalitis in humans. This is illustrated by mutations in TBK1, IRF3, or Toll-like receptor 3 (TLR3) in individual patients [72–75]. Consistently, in murine HSV encephalitis models, deficiency in MAVS or in TRIF that is a TLR3 adaptor, increases mortality rates [76]. It is tempting to speculate that inhibition of RIG-I by γ_1_34.5 may favor HSV-mediated pathology. Further development of relevant *in vivo* models will be required to address this question. Moreover, genetically modified HSV that lacks γ_1_34.5 is avirulent and approved for melanoma therapy in humans [21,24]. Recent work further suggested that its combination with immune checkpoint blockade enhances therapeutic efficacy [23]. Therefore, it would be intriguing to investigate whether RIG-I activation by γ_1_34.5 null oncolytic HSV primes antitumor immunity. Further characterization may lead to the development of next generation therapeutic agents.

## Materials and methods

### Cells and viruses

Vero, HEK-293T, Human lung embryonic (HEL) fibroblasts cells were obtained from the American Type Culture Collection. RIG-I (Ddx58) wild type (*Rig-I*^*+/+*^) MEFs and RIG-I knockout (*Rig-I*^*-/-*^ MEFs, MDA5 (Ifih1) wild type (*Mda5*^*+/+*^) MEFs and MDA5 knockout (*Mda5*^*-/-*^) were described previously [77]. HEL stably expressed Non-Target shRNA (shCtrl) or RIG-I target shRNA (shRIG-I) were selected with puromycin (sc-205821, Santa Cruz Biotechnology) at the concentration 3μg/ml. Cells were propagated in Dulbecco’s modified Eagle’s medium (DMEM) supplemented with 5% or 10% fetal bovine serum. HSV-1(F) is a prototype HSV-1 strain used in this study [78]. In recombinant virus Δγ_1_34.5, a 1-kb fragment from the coding region of the γ_1_34.5 gene was deleted [60]. In recombinant virus HSV-1(R), the deleted coding region of γ_1_34.5 was repaired to restore the wild type γ_1_34.5 gene [60]. Preparation of viral stock and titration of infectivity were carried out with Vero cells.

### Antibodies

Mouse anti-β-actin (A5316) and anti-Flag-HRP antibodies (A8592) were purchased from Sigma-Aldrich. Anti-Myc-horseradish peroxidase (HRP) antibody (#2040), anti-HA-HRP antibody (#2999), rabbit anti-IRF-3 (#11904), rabbit anti-pIRF-3 (#4947), rabbit anti-Flag (#14793), rabbit anti-LDHA (#3582), rabbit anti-COX IV (#4844), anti-rabbit IgG-HRP-linked antibody (#7074), anti-mouse IgG-HRP-linked antibody (#7076), normal rabbit IgG (#2729) were bought from Cell-Signaling Technologies. Mouse anti-Myc antibody (sc-40), mouse anti-HA antibody (sc-7392), mouse anti-RIG-I (sc-376845), mouse anti-14-3-3ε-horseradish peroxidase (HRP) antibody (sc-23957 HRP), mouse anti-V5 antibody (sc-81594), mouse anti-MAVS (sc-365334), normal mouse IgG (sc-2025) were purchased from Santa Cruz Biotechnology. Mouse anti-HSV ICP27 antibody (P1113) was purchased from Virusys. Anti-γ_1_34.5 and NS1 antibodies were described previously [79,80].

### Plasmids and reporter assays

pLKO.1-puro Non-Target shRNA Control Plasmid and pLKO.1-puro RIG-I Target shRNA Plasmid were purchased from Sigma-Aldrich. The lentivirus package plasmids pCMV-VSV-G, pMDLg/pRRE, and pRSV-REV were previously described [81]. Flag-RIG-I, Myc-RIG-I, Myc-RIG-I-2CARDs, Myc-14-3-3ε, Flag-NS3-Pro (DENV), V5-TRIM25, HA-Ub (K63 only), Flag-γ_1_34.5, Flag-N159, Flag-ΔN146, Flag-mCherry and pCAGGS-NS1 were described previously [6,44,53,82–84]. For luciferase reporter assays, HEK-293T cells grown in 24-well plates were transfected with a luciferase reporter plasmid IFN-β-Luc, and pRL-TK (Promega), together with the indicated expression plasmid or an empty vector using Lipofectamine 3000 (Invitrogen) according to the manufactural instruction. At 48 h posttransfection, cells were harvested for Dual-Luciferase reporter assay system [14]. In Sendai virus infection experiments, cells were stimulated with the virus (100 HA/ml) for 24 h.

### Virus infection assay

Cells were infected with viruses at the indicated multiplicity of infection. After adsorption for 2 h, the monolayers were overlaid with DMEM supplemental with 1% FBS and incubated at 37°C. For viral titer determination, samples were harvested at 48 hours postinfection and viruses, released by three cycles of freezing and thawing, were titrated on Vero cells [53].

### RNA sequencing and data analysis

Total RNA from Mock, HSV-1(F) and Δγ_1_34.5 infected MEFs was extracted using a RNeasy Plus mini kit (Qiagen) and then subjected to RNA-deep sequencing (RNA-seq) analysis (Novogene). Sequencing libraries were generated using NEBNext Ultra RNA Library Prep Kit for Illumina following the manufacturer’s recommendations, and index codes were added to attribute sequences to each sample. The clustering of the index-coded samples was performed on a cBot Cluster Generation System using TruSeq PE Cluster Kit v3-cBot-HS (Illumina). After cluster generation, the library preparations were sequenced on an Illumina HiSeq platform, and 125 bp/150 bp paired-end reads were generated.

Raw reads were aligned to the mouse reference genome in a splice-aware manner using the STAR aligner [85]. ENSEMBL gene and transcript annotations, which include non-coding RNAs in addition to mRNAs, were used. With Feature Counts [86], gene expression was first quantified as raw read counts and then normalized to reads-per-million for direct comparison between samples. Pair-wise differential expression statistics (fold-change and p-value) were computed using edgeR [87,88]. p-values were adjusted for multiple testing using the false discovery rate (FDR) correction of Benjamini and Hochberg [89].

To perform GSEA analysis on RNA-seq datasets, the log fold changes of all genes in edgeR result output were used to generate a ranked list for GSEA preranked analysis using the Molecular Signatures Database v5.2 (H: hallmark gene sets) [35]. Specifically, the differences in log2 fold changes of all genes in virus-infected cells relative to the mock group were used to generate the ranked list. Enriched gene sets ranked by GSEA normalized enrichment score (NES) were visualized using ggplot2 package in R. Gene sets with a nominal p value < 0.05 and false discovery rate (FDR) < 0.25 were defined as significantly enriched. Heat maps were produced from the primary data (the normalized expression value) using the R package pheatmap v1.0.8.

### Quantitative real-time PCR assay

Total RNA was harvested from cells using a RNeasy Plus mini kit (Qiagen). Genomic DNA was eliminated using gDNA Eliminator columns. cDNA was synthesized using a high-capacity cDNA reverse transcription kit (Applied Biosystems). Quantitative real-time PCR was performed using an Applied Biosystems ABI Prism 7900HT instrument with SYBR green master mix (Applied Biosystems). Gene expression levels were normalized to that of endogenous control 18S rRNA. Relative gene expression was determined as described previously [90]. All primers were listed in S1 Table.

### Western blot

Cells were harvested, washed with phosphate-buffered saline (PBS), and lysed as described previously [91]. Samples were then subjected to electrophoresis on denaturing polyacrylamide gels, transferred to Polyvinylidene difluoride (PVDF) membranes, and reacted with indicated antibodies [83].

### Immunoprecipitation and ubiquitination analysis

To detect protein interactions, immunoprecipitation was performed as described previously [91]. Briefly, cells were lysed, and cell extracts were incubated with the indicated antibodies and agarose conjugated with protein A/G (sc-2003, Santa Cruz Biotechnology) at 4°C. The beads were washed three times with wash buffer (50 mm Tris-HCl, pH 7.4, 150 mm NaCl, 5 mm EDTA, 0.1% Triton X-100, and protease inhibitor mixture). The samples were then subjected to immunoblotting analysis. For detection of RIG-I ubiquitination, cells were lysed with lysis buffer (1% SDS, 150 mM NaCl, 10 mM Tris-HCl, pH 8.0) with 2mM sodium orthovanadate, 50 mM sodium fluoride, and protease inhibitors. Samples were precipitated and washed RIPA buffer containing 2M urea to remove nonspecific binding of other ubiquitinated proteins.

### Lentiviral transduction

pLKO.1 Puro RIG-I Target shRNA Plasmid and pLKO.1 Puro Non-Target shRNA Control Plasmid (negative control) were purchased from shRNA (Sigma-Aldrich). The lentivirus was produced after transfection of shRNA plasmid together with package plasmids (pCMV-VSV-G, pMDLg/pRRE, and pRSV-REV) in HEK-293T cells. HEL were then infected with the collected lentivirus. At 16 h after infection, the medium was replaced with fresh medium. At 3 days after infection, the cells were selected by 3μg/ml puromycin (sc-205821, Santa-Cruz Biotechnology). Experiments were performed within 2 weeks after lentiviral transduction.

### Mitochondrial fractionation analysis

MEFs were mock infected or infected with wild type HSV-1(F) and Δγ_1_34.5. At 8 h postinfection, the cells were harvested to prepare cytoplasmic and mitochondrial fractions using an EzSubcell Fraction Kit (ATTO, Tokyo, Japan). Samples were then analyzed by immunoblotting. For HEK-293T analysis, cells were transfected with Flag-γ_1_34.5 for 24 h, followed by treatment with SeV at the 100HA/ml for additional 24h. And the cells were harvested for cell fractionation analysis.

### Statistical analysis

All data were presented as means ± SD and analyzed using GraphPad Prism software (version 6). One-way ANOVA with Dunnett’s multiple comparisons or an unpaired two-tailed Student’s t test was used as indicated in the legends. For the graphs, data were in general three biological replicates and reproduced in independent experiments as indicated in the legends.

## Supporting information

S1 Fig Pathway analysis with GSEA. GSEA of RNA-seq data showing significantly enriched hallmark signatures of IFN-α (A) and IFN-γ (B) pathways in virus-infected cells. The plots compare enrichment scores between cells infected with the γ_1_34.5 null virus and wild type HSV-1 as described in MATERIALS AND METHODS.
(TIF)

S2 Fig Inhibition of RIG-I dependent antiviral responses by the γ_1_34.5 gene product is not due to an irrelevant mutation(s) elsewhere in the HSV-1 genome. (A) Effects of γ_1_34.5 on antiviral gene expression in *Rig-I*^*+/+*^ or *Rig-I*^*-/-*^ MEF cells. Cells, infected with HSV-1, Δγ_1_34.5 or its repair virus HSV-1(R) (5 pfu/cell) for 8 h, were analyzed for transcript levels of IFN-β, Ifit1, Ifit2, and Ccl5 by quantitative PCR analysis. The data were statistically analyzed by one-way ANOVA (**, *P* < 0.01), with standard deviations (SD) (n = 3). (B) Effects of γ_1_34.5 on IRF3 phosphorylation in RIG-I^+/+^ or RIG-I^-/-^ MEF cells. Cells were infected as described in panel A and processed for western blot analysis with antibodies against p-IRF3, IRF3, ICP27, γ_1_34.5, RIG-I and β-actin. The experimental data are representative of results from three independent experiments.
(TIF)

S3 Fig The γ_1_34.5 protein interacts with RIG-I CARD domain and inhibits RIG-I induced IFN-β promoter activity. (A) HSV-1 γ_1_34.5 binds the RIG-I CARD domain. HEK-293T cells were transfected with Myc-RIG-I-2CARDs together with empty vector (Vec) or Flag-γ_1_34.5 or Flag-mCherry for 36 h. Whole-cell lysates (WCLs) were subjected to immunoprecipitation (IP) with anti-Myc antibody. Precipitated proteins and whole-cell lysates (WCL) were probed with antibodies against Flag, Myc, and β-actin. (B) The γ_1_34.5 protein inhibits IFN-β promoter activation by RIG-I. HEK-293T cells were co-transfected with Myc-RIG-I-2CARDs (100 ng), pIFN-β-luc (50 ng) and pRL-TK (10 ng) along with the Vector (400ng) or Flag-γ_1_34.5(400ng) or pCAGGS-NS1(400ng). At 48 h after transfection, luciferase activities were determined. (C) The γ_1_34.5 protein inhibits RIG-I in a dose dependent manner. HEK-293T cells were co-transfected with different doses of Flag-γ_1_34.5 and harvested for luciferase assays as described in (B). Results are expressed as fold activation relative to the empty vector control with SD (*n* = 3) and assessed by one-way ANOVA (**, *P* < 0.01) for (A) and (B). The experimental data are representative of results from three independent experiments.
(TIF)

S4 Fig Intact γ_1_34.5 is required to interact with and inhibit RIG-I. (A) Schematic depiction of the γ_1_34.5 variants. Numbers indicate amino acid positions. (B) and (C) The γ_1_34.5 protein interacts with RIG-I in the absence of other viral proteins. HEK-293T cells were transfected with plasmids encoding Myc-RIG-I together with empty vector (Vec) or Flag-tagged γ_1_34.5 variants (γ_1_34.5, ΔN146 and N159) for 36 h. Whole-cell lysates (WCLs) were subjected to immunoprecipitation (IP) with anti-Myc (B) or anti-Flag (C) antibody. Precipitated proteins and whole-cell lysates (WCL) were probed with antibodies against Flag, Myc, and β-actin. (D) Effects of γ_1_34.5 variants on IFN-β promoter activation by the RIG-I-2CARDs domain. HEK-293T cells were co-transfected with Myc-RIG-I-2CARDs (100 ng), pIFN-β-luc (50 ng) and pRL-TK (10 ng) along with the Vector, Flag- γ_1_34.5 or its mutants (Flag-ΔN146 and Flag-N159). Cells were harvested for luciferase assays at 48 h after transfection. Results are expressed as fold activation relative to the empty vector control with SD (*n* = 3) and assessed by one-way ANOVA (**, *P* < 0.01). The experimental data are representative of results from three independent experiments.
(TIF)

S5 Fig The γ_1_34.5 protein inhibits RIG-I-14-3-3ε complex translocation to the mitochondria. The influence of γ_1_34.5 gene on RIG-I and 14-3-3ε mitochondrial localization after SeV stimulation. HEK-293T cells were transfected with Flag-γ_1_34.5 for 24 h, which was followed by SeV stimulation at the 100 HA/ml for additional 24 h. Cells were harvested and analyzed for the RIG-I and 14-3-3ε in the cytoplasmic and mitochondrial fractions. The experimental data are representative of results from three independent experiments.
(TIF)

S6 Fig MDA5 is not associated with the replication of γ_1_34.5 null mutant HSV-1. (A) Viral replication in *Mda5*^*+/+*^ or *Mda5*^*-/-*^ MEFs. Cells were infected with wild-type HSV-1 and the γ_1_34.5 deletion virus (Δγ_1_34.5) at a MOI 0.01. At 48 h postinfection, the total virus yields were determined on Vero cells using plaque assay. (B) Kinetics of viral growth in *Mda5*^*+/+*^ or *Mda5*^*-/-*^ MEFs. Viral infection was performed as described in panel (A) and viral yields were measured at indicated time points. The data are representative of results from three experiments with triplicate samples. Differences between the selected groups were statistically assessed by one-way ANOVA for (A) or a two-tailed Student’s t test for (B) (**, *P* < 0.01).
(TIF)

S1 Table Primers used for RT-PCR. (XLSX)
