# Supplementary figures and images for: The herpesvirus accessory protein γ134.5 facilitates viral replication by disabling mitochondrial translocation of RIG-I
Source: PLoS Pathog. 2021 Mar 26;17(3):e1009446. doi: 10.1371/journal.ppat.1009446 (PMC7996975; doi:10.1371/journal.ppat.1009446)

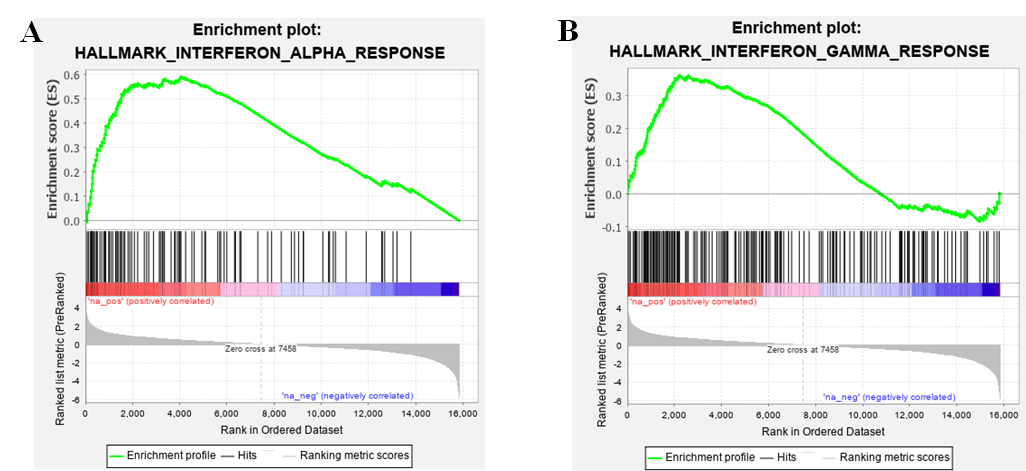

Supplement: S1 Fig — GSEA of RNA-seq data showing significantly enriched hallmark signatures of IFN-α (A) and IFN-γ (B) pathways in virus-infected cells. The plots compare enrichment scores between cells infected with the γ134.5 null virus and wild type HSV-1 as described in MATERIALS AND METHODS. (TIF) [file ppat.1009446.s001.tif]

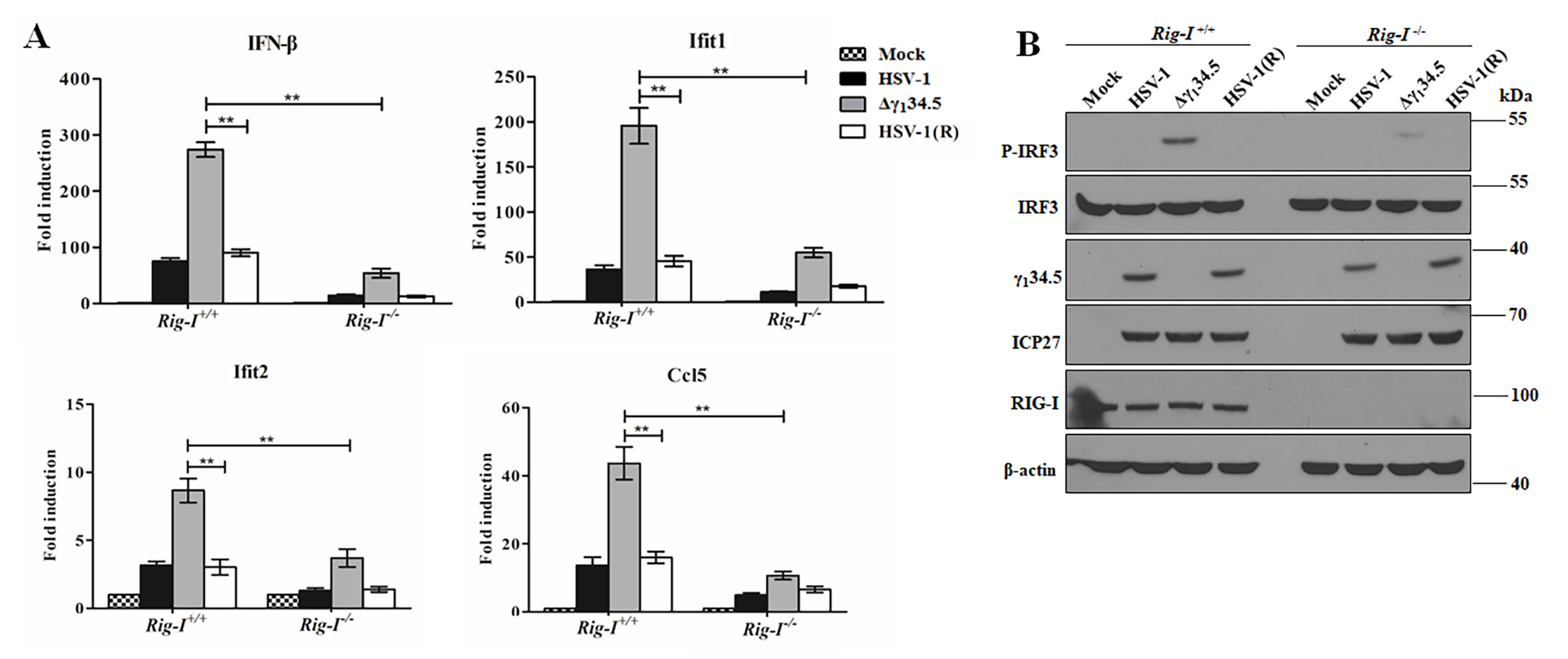

Supplement: S2 Fig — (A) Effects of γ134.5 on antiviral gene expression in Rig-I+/+ or Rig-I-/- MEF cells. Cells, infected with HSV-1, Δγ134.5 or its repair virus HSV-1(R) (5 pfu/cell) for 8 h, were analyzed for transcript levels of IFN-β, Ifit1, Ifit2, and Ccl5 by quantitative PCR analysis. The data were statistically analyzed by one-way ANOVA (**, P < 0.01), with standard deviations (SD) (n = 3). (B) Effects of γ134.5 on IRF3 phosphorylation in RIG-I+/+ or RIG-I-/- MEF cells. Cells were infected as described in panel A and processed for western blot analysis with antibodies against p-IRF3, IRF3, ICP27, γ134.5, RIG-I and β-actin. The experimental data are representative of results from three independent experiments. (TIF) [file ppat.1009446.s002.tif]

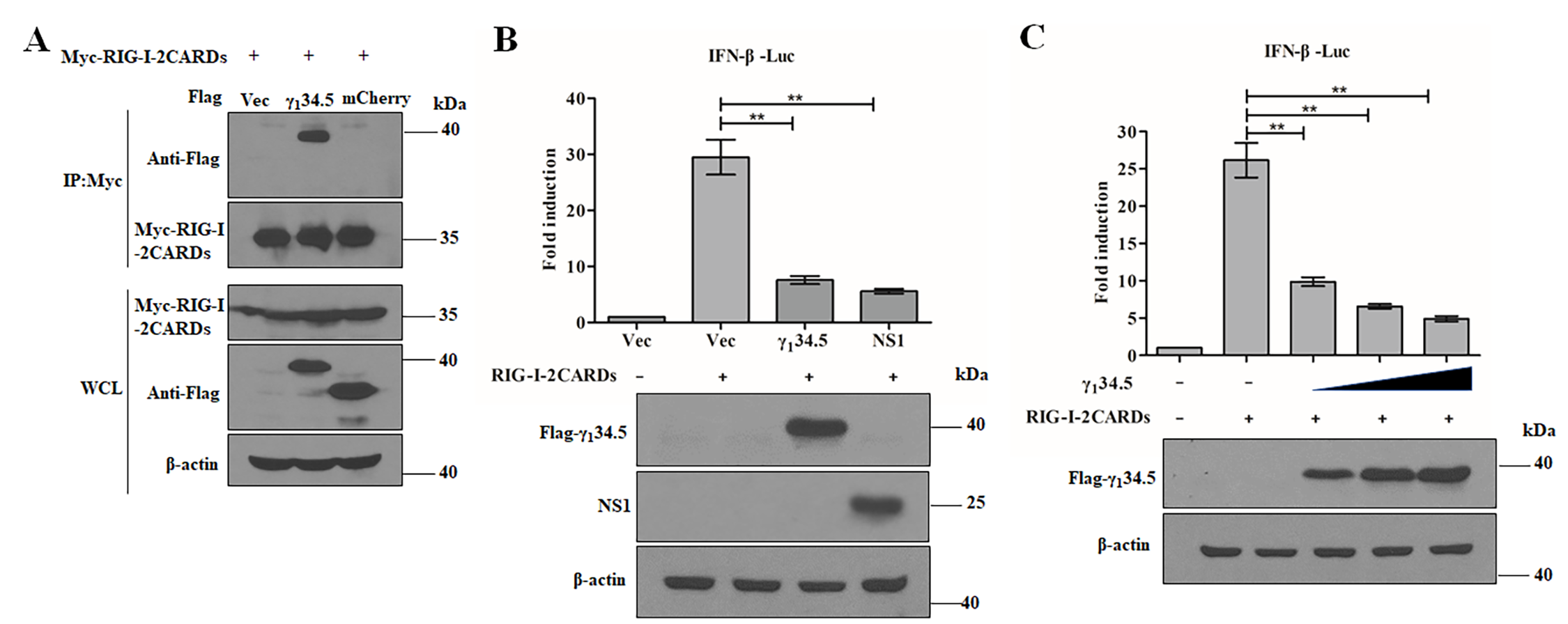

Supplement: S3 Fig — (A) HSV-1 γ134.5 binds the RIG-I CARD domain. HEK-293T cells were transfected with Myc-RIG-I-2CARDs together with empty vector (Vec) or Flag-γ134.5 or Flag-mCherry for 36 h. Whole-cell lysates (WCLs) were subjected to immunoprecipitation (IP) with anti-Myc antibody. Precipitated proteins and whole-cell lysates (WCL) were probed with antibodies against Flag, Myc, and β-actin. (B) The γ134.5 protein inhibits IFN-β promoter activation by RIG-I. HEK-293T cells were co-transfected with Myc-RIG-I-2CARDs (100 ng), pIFN-β-luc (50 ng) and pRL-TK (10 ng) along with the Vector (400ng) or Flag-γ134.5(400ng) or pCAGGS-NS1(400ng). At 48 h after transfection, luciferase activities were determined. (C) The γ134.5 protein inhibits RIG-I in a dose dependent manner. HEK-293T cells were co-transfected with different doses of Flag-γ134.5 and harvested for luciferase assays as described in (B). Results are expressed as fold activation relative to the empty vector control with SD (n = 3) and assessed by one-way ANOVA (**, P < 0.01) for (A) and (B). The experimental data are representative of results from three independent experiments. (TIF) [file ppat.1009446.s003.tif]

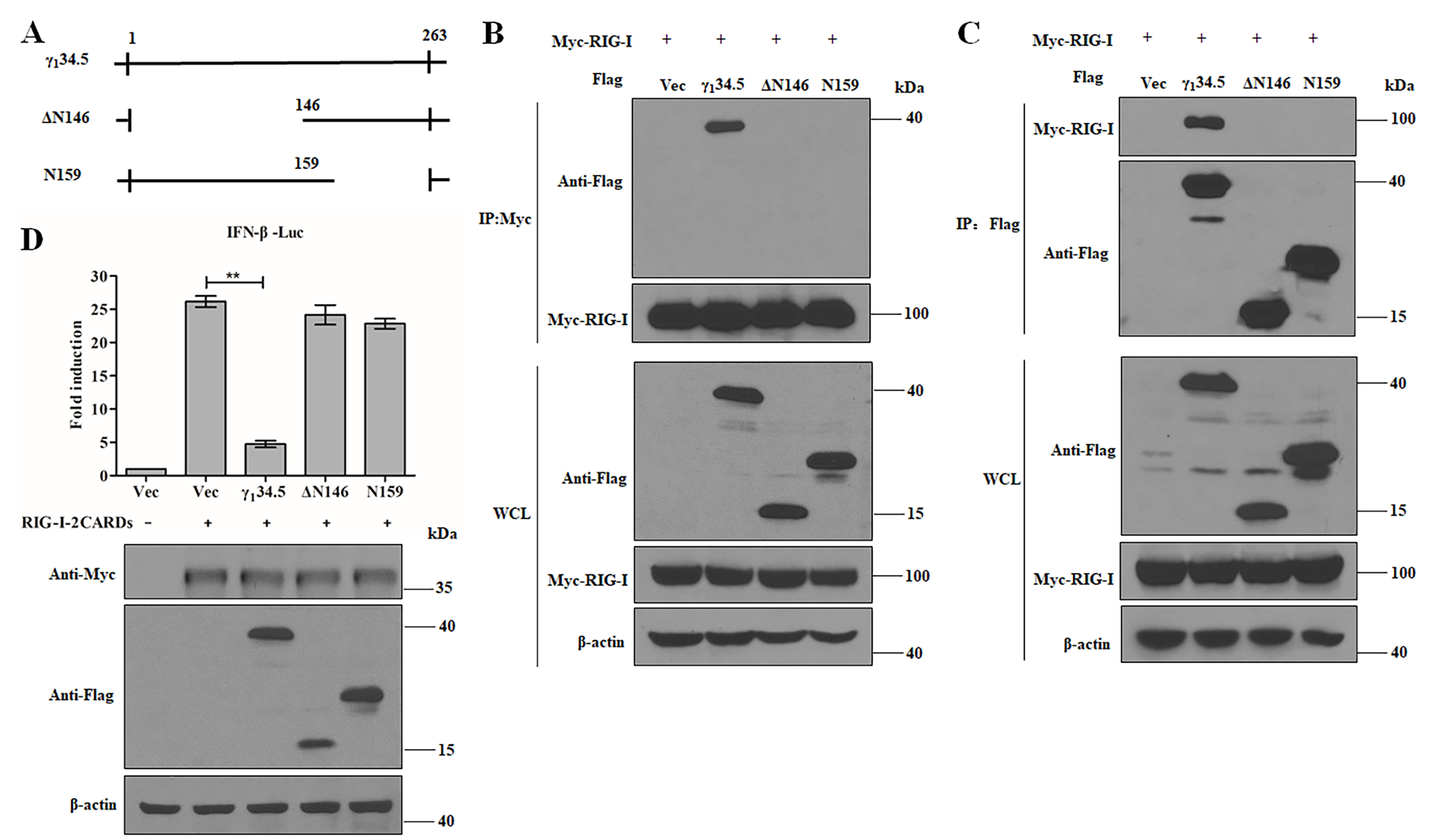

Supplement: S4 Fig — (A) Schematic depiction of the γ134.5 variants. Numbers indicate amino acid positions. (B) and (C) The γ134.5 protein interacts with RIG-I in the absence of other viral proteins. HEK-293T cells were transfected with plasmids encoding Myc-RIG-I together with empty vector (Vec) or Flag-tagged γ134.5 variants (γ134.5, ΔN146 and N159) for 36 h. Whole-cell lysates (WCLs) were subjected to immunoprecipitation (IP) with anti-Myc (B) or anti-Flag (C) antibody. Precipitated proteins and whole-cell lysates (WCL) were probed with antibodies against Flag, Myc, and β-actin. (D) Effects of γ134.5 variants on IFN-β promoter activation by the RIG-I-2CARDs domain. HEK-293T cells were co-transfected with Myc-RIG-I-2CARDs (100 ng), pIFN-β-luc (50 ng) and pRL-TK (10 ng) along with the Vector, Flag- γ134.5 or its mutants (Flag-ΔN146 and Flag-N159). Cells were harvested for luciferase assays at 48 h after transfection. Results are expressed as fold activation relative to the empty vector control with SD (n = 3) and assessed by one-way ANOVA (**, P < 0.01). The experimental data are representative of results from three independent experiments. (TIF) [file ppat.1009446.s004.tif]

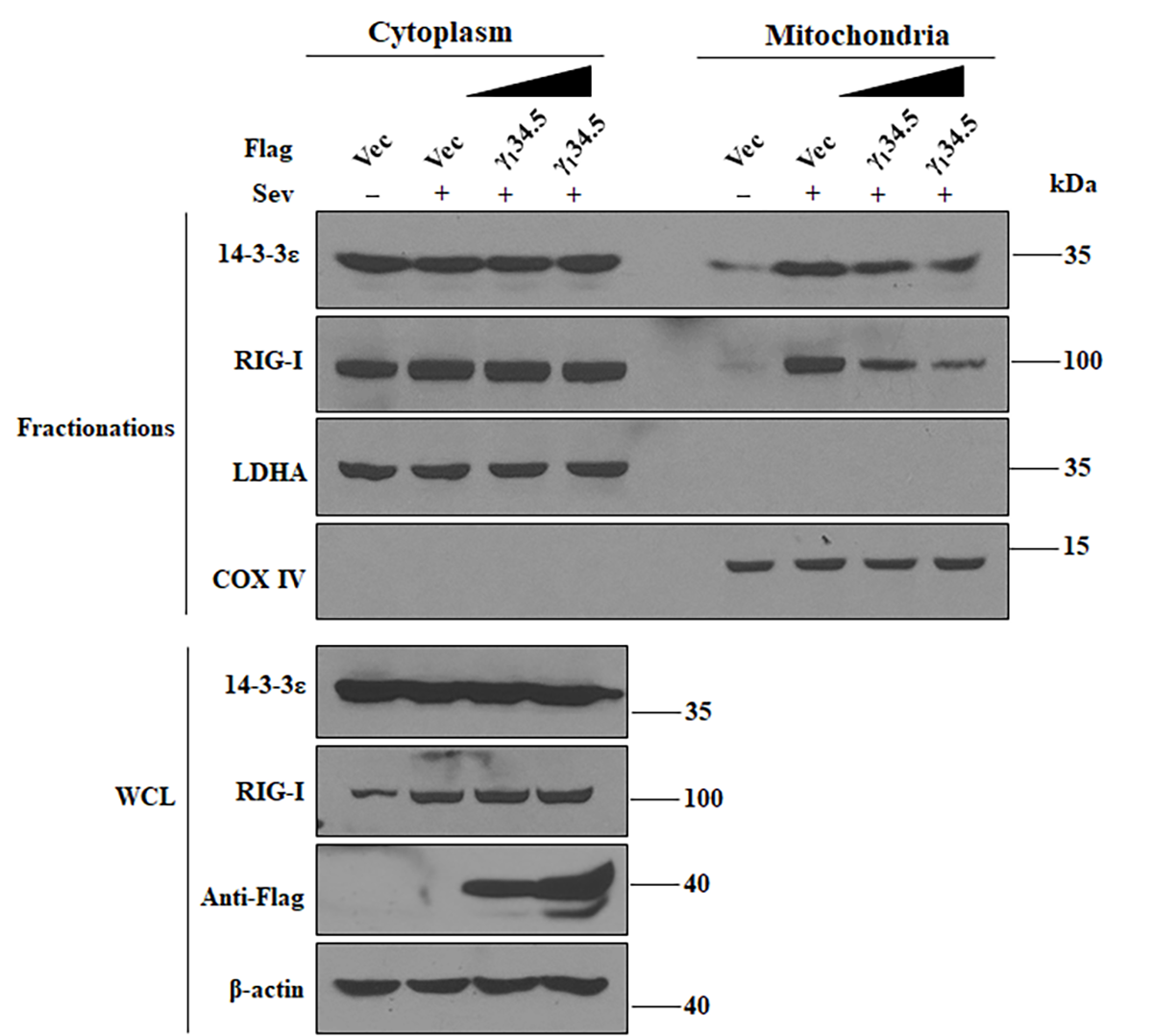

Supplement: S5 Fig — The influence of γ134.5 gene on RIG-I and 14-3-3ε mitochondrial localization after SeV stimulation. HEK-293T cells were transfected with Flag-γ134.5 for 24 h, which was followed by SeV stimulation at the 100 HA/ml for additional 24 h. Cells were harvested and analyzed for the RIG-I and 14-3-3ε in the cytoplasmic and mitochondrial fractions. The experimental data are representative of results from three independent experiments. (TIF) [file ppat.1009446.s005.tif]

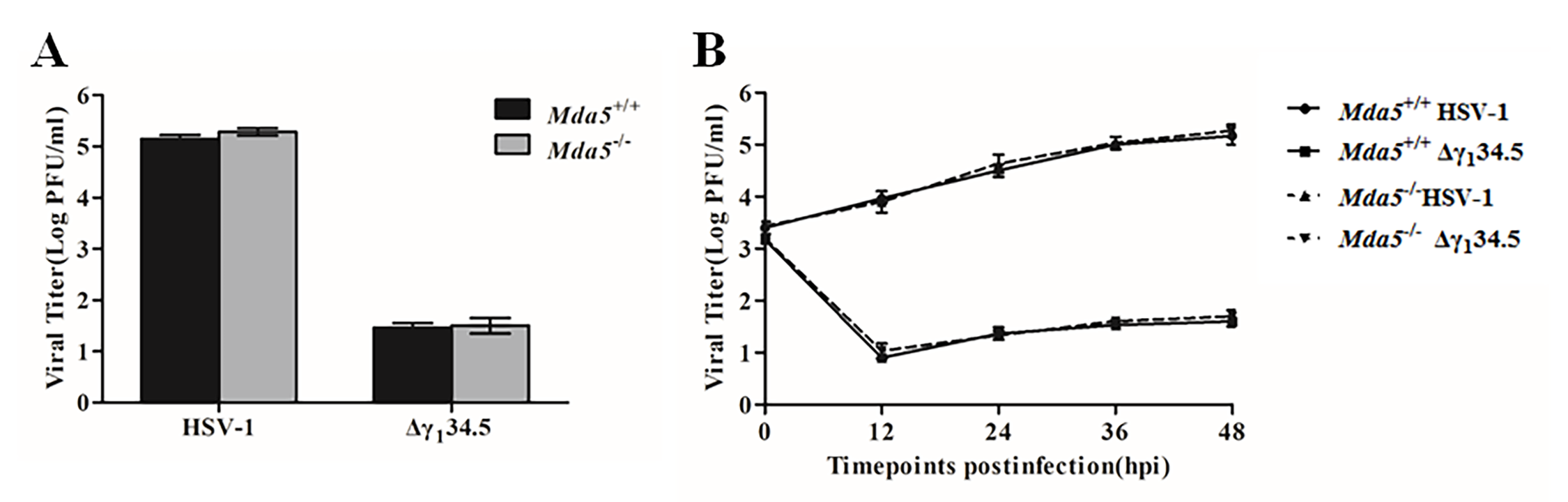

Supplement: S6 Fig — (A) Viral replication in Mda5+/+ or Mda5-/- MEFs. Cells were infected with wild-type HSV-1 and the γ134.5 deletion virus (Δγ134.5) at a MOI 0.01. At 48 h postinfection, the total virus yields were determined on Vero cells using plaque assay. (B) Kinetics of viral growth in Mda5+/+ or Mda5-/- MEFs. Viral infection was performed as described in panel (A) and viral yields were measured at indicated time points. The data are representative of results from three experiments with triplicate samples. Differences between the selected groups were statistically assessed by one-way ANOVA for (A) or a two-tailed Student’s t test for (B) (**, P < 0.01). (TIF) [file ppat.1009446.s006.tif]
